# NLRscape: an atlas of plant NLR proteins

**DOI:** 10.1093/nar/gkac1014

**Published:** 2022-11-09

**Authors:** Eliza C Martin, Catalin F Ion, Florin Ifrimescu, Laurentiu Spiridon, Jaap Bakker, Aska Goverse, Andrei-J Petrescu

**Affiliations:** Department of Bioinformatics and Structural Biochemistry, Institute of Biochemistry of the Romanian Academy, Bucharest 060031, Romania; Department of Bioinformatics and Structural Biochemistry, Institute of Biochemistry of the Romanian Academy, Bucharest 060031, Romania; Department of Bioinformatics and Structural Biochemistry, Institute of Biochemistry of the Romanian Academy, Bucharest 060031, Romania; Department of Bioinformatics and Structural Biochemistry, Institute of Biochemistry of the Romanian Academy, Bucharest 060031, Romania; Laboratory of Nematology, Wageningen University and Research, Wageningen 6700ES, The Netherlands; Laboratory of Nematology, Wageningen University and Research, Wageningen 6700ES, The Netherlands; Department of Bioinformatics and Structural Biochemistry, Institute of Biochemistry of the Romanian Academy, Bucharest 060031, Romania

## Abstract

NLRscape is a webserver that curates a collection of over 80 000 plant protein sequences identified in UniProtKB to contain NOD-like receptor signatures, and hosts in addition a number of tools aimed at the exploration of the complex sequence landscape of this class of plant proteins. Each entry gathers sequence information, domain and motif annotations from multiple third-party sources but also in-house advanced annotations aimed at addressing caveats of the existing broad-based annotations. NLRscape provides a top-down perspective of the NLR sequence landscape but also services for assisting a bottom-up approach starting from a given input sequence. Sequences are clustered by their domain organization layout, global homology and taxonomic spread—in order to allow analysis of how particular traits of an NLR family are scattered within the plant kingdom. Tools are provided for users to locate their own protein of interest in the overall NLR landscape, generate custom clusters centered around it and perform a large number of sequence and structural analyses using included interactive online instruments. Amongst these, we mention: taxonomy distribution plots, homology cluster graphs, identity matrices and interactive MSA synchronizing secondary structure and motif predictions. NLRscape can be found at: https://nlrscape.biochim.ro/.

## INTRODUCTION

Intracellular Nod-like receptors (NLR) play a key role in plant immunity by activating the response leading to plant disease resistance. As being involved either directly in pathogen effector recognition or in monitoring the modifications induced by these onto the host targets—NLRs and NLR networks were always at the forefront of the battle against pathogens and pests which have driven their high gene diversification and the high dynamics of disease-resistance loci in plant genomes (1,2). Given their importance in disease resistance and pest control NLRs are arguably one of the most studied plant protein families with a wealth of public resources gathering information on their location and properties such as PRGdb or RefPlantNLR (3–5).

Also known as resistance R proteins, they contain a central nucleotide-binding domain which acts as an on/off activation switch, followed by a solenoid domain, most often a leucine-rich repeat (LRR) domain and more rarely an armadillo, ankirin, thioredoxin (TRX), tetratricopeptide (TPR), WD or kelch, etc. (6,7). Based on the presence of a distinct N-terminal domain NLRs classify into four main classes : (i) CNL which contains a coiled-coil (CC) domain, (ii) TNL containing a Toll/interleukin-1 receptor (TIR) domain, (iii) RNL with a Resistance To Powdery Mildew 8 (RPW8) domain or (iv) NL—which do not encode a canonical N-ter domain (7,8). Besides these main class outlines, a large plethora of non-canonical integrated domains (ID) acting as decoys that mimic pathogen protein targets has been intensively studied (6,9–11).

Whereas in the last two decades, the research of this complex class of proteins was hindered by having at hand only remote homology models (12–20) and a handful of 3D structures of individual domains in different stages of the mechanism, some of which with conflicting structures (21–27), a major advance in the field stood in solving the full-protein cryo-EM structure of ZAR1 in resting state and pentameric activated state (28,29), followed shortly by the cryo-EM structure of Roq1 and Rpp1 tetrameric TNL complexes (30,31). These new data were instrumental in providing detailed insights into the NLR’s intricate biological mechanisms, while also underlying their high diversity and functional complexity (32,33).

Proof of concept experiments of comparative-sequence pipelines and structural-guided NLR engineering have succeeded in extending the native NLR recognition spectrum of given receptors to a novel pathogen effector (34–36). Such advances highlight the importance of employing both structurally-informed interventions and exploiting the highly dynamic variation naturally occurring—even within individuals of the same species (37,38)—in designing experimental interventions to expand our understanding of the complex NLR landscape and to allow the development of crop resistance control technologies with vast applications in a pesticide-free agriculture (39,40).

While the existing public resources such as PRGdb, RefPlantNLR (4,5), focus mainly on genomic data or reference datasets and the identification of new NLR genes providing general information and annotations for disease resistance analysis, little emphasis is directed at the time being in the public domain on an in-depth structural description and annotation of the so far identified NOD-like receptors—which is critical in the investigation of structure-function relations and structure-guided engineering of plant immunity.

In this context the herein introduced web server NLRscape is intended to complement the existing resources and aid structural analysis by creating an online environment which, besides being a data hub for the NLR protein family, would feature both an in-depth overview of the NLR sequence landscape, but also facilitate homology-based inferences onto the structure-function relations in newly discovered sequences. Hence, in brief, NLRscape is an interactive online atlas of plant NLRs—designed to aid: (i) structure-informed analysis for intervention design; (ii) investigation of NLR evolutionary dynamics and speciation; (iii) pattern analysis; (iv) artificial evolution attempts to expand NLR diversity via bioengineering—given the documented importance of cluster variability profile analysis combined with cumulated information on NLR motifs, 3D modelling and sequence to structure mapping in such kind of studies (34,36–38,41–43).

## MATERIALS AND METHODS

### The database assembly

A collection of 80 303 potential plant NLR candidates was gathered from UniProtKB (44) by selecting proteins that contain NBS activation switch domains, as these are central to all NLR classes. A preliminary *in-house* annotation was performed employing a profile hidden Markov model (HMM) screening using HMMER (45). The queried HMM profiles were generated starting from an initial collection of highly diverse NBS representatives gathered via sequence clustering of the previously annotated NBS/NB-ARC/NACHT domains currently present in the Interpro collection (46) using MMseqs2 (47) with the greedy set cover clustering method and a bidirectional 70% overlap and 30% identity cutoffs. The sequences gathered were further subjected to a more precise annotation process to delineate the NBS constituent subunits, other NLR-associated domains such as CC, TIR, RPW8, LRR, etc. and identify NLR-specific sequence motifs.

### Annotation of canonical NLR domains and motifs

#### NBS domain annotations

A preliminary analysis of the available Interpro annotations was performed to assess the existing coverage on the gathered set. While all the selected sequences were supported by at least one annotation in the Interpro collection for at least one NBS/NB-ARC/NACHT-related tag, domain and subdomain range annotations originating from various sources revealed conflictual and/or inconsistent delineations, especially at the borders of the NBS subunits, CC and LRR domains. Conflictual entries were reduced to a unique annotation via merging criteria. The resulting refined annotations were evaluated for consistency especially at domain borders, while sequence regions larger than 50 residues not being covered by existing annotations were investigated to assess if they correspond to NLR-related domains.

The NBS regions identified in the sequence data set were further analyzed by delineating their individual subunits in order to achieve a more detailed domain mapping and enrich the annotation. To this end, query HMM profiles of the NBD, ARC1 and ARC2 subdomains were built using a trimmed representative set of sequences obtained by sequence clustering using MMseqs2 (47) at 30% identity and 70% overlap cutoffs. Cluster representatives were further manually curated to eliminate incomplete sequences and examine the sequence compliance to the subdomain secondary structure profile—needed to avoid wrong assignments induced by the high sequence variability of the inter-subunit regions shown to inflict on the overall multiple sequence alignments.

#### CC/TIR/RPW8 domain annotations

The NLR region upstream of the NBS domain exhibits a much higher variability compared to NBS and all other NLR-associated domains in general. Three main sequence groups were described in this region known as the CC, TIR and RPW8 canonical domains (8). While TIR and RPW8 are consistently homogenous, the CC group is highly variable and displays a very broad spectrum of lengths. This results in stark inconsistencies of Interpro CC annotations, in contrast to those of TIR and RPW8. When not completely absent as in the case of arabidopsis RPS2 and RPS5—known experimentally to display CC domains (48), annotations were shown to display severe border problems, especially in locating the first and last predicted helical segments. In order to refine the CC domain delineation the following iterative workflow was used: in a first step a number of 32 799 CC domains already annotated in Interpro were clustered at 30% identity; in the second step selected cluster representatives were used to generating an overall HMM profile which was used to detect putative CC domains in NLRscape dataset with domain e-values above 0.01; in the third step the 35 220 identified potential domains were reclustered at the same sequence identity threshold and the resulting representatives were profiled for secondary structure propensity to avoid false positives. In this way, a total of 110 highly divergent clusters were selected to generate 110 HMM profiles each describing distinct CC classes <30% identity. These profiles were used to rescreen the NLRscape collection and identify a total of 45 429 putative CC domains, which is an increase of ∼39% compared to the available annotations in the Interpro database.

#### LRR domain annotations

In canonical NLRs, the region downstream NBS is known as the LRR domain. This is rich in *LxxLxL* motifs that generate a repetitive solenoidal ‘horseshoe’ scaffold held together by hydrogen bonds between consecutive motifs (49). Interpro loosely annotates both overall LRR domains and some of the motifs considered unambiguous, scantily spread over the entire defined domain. In order to improve both domain and motif annotations, we used LRRpredictor shown to locate LRR motifs with over 90% accuracy (50). Based on the predicted LRR motifs we concatenated individual LRR repeats whenever at least four motifs were annotated successively in a sequence. Identified inter-motif regions longer than 75 residues were considered potential interruptions of the LRR scaffold and consequently the two LRR regions were annotated as consecutive LRR domains separated by a linker.

#### Non-canonical, less frequent domain annotations

Additional less frequently encountered sequence regions such as decoy or signalling domains (jacalin, late blight, WRKY, etc.) or solenoid domains (armadillo, tetratricopeptide, WD40) identified in NLRscape dataset, were scrutinized for consistency with Interpro domain annotations. As the detected inconsistencies were significantly less frequent providing custom annotations was not considered necessary.

#### Annotations of main motifs in canonical NLR domains

NLR-specific sequence motifs – such as the EDVID, found in many CC domains, the β/α A–D motifs specific to TIR domains and the nucleotide-binding or intramolecular contact motifs specific to NBS domains (P-loop, Walker-B, RNBS A/B/C/D, GLPL, MHD) were identified using NLRexpress (51), while and the LRR motifs were predicted using LRRpredictor (50).

#### NBS domain—functionality scoring

As activation is a key NLR function and this depends on the NBS domain integrity and its ability to switch between inactive and active state (8), a 6-state scoring system was introduced to assess NBS functionality of each NLRscape entry. This is based on the presence of 15 highly conserved nucleotide contact residues identified from ZAR1 & RPP1 CNL/TNL structures (28–30) shown in Supplementary Table S1 and the identification of nine key functional motifs involved in the formation of the nucleotide binding pocket and 3D structural stability of NBS using NLRexpress (51): VG/bbGRE, P-loop/Walker-A, RNBS -A, -B, -C and -D, GLPL.

The system runs as follows: (A) ‘Highly likely functional’—comprising all motifs and conserved contacts, (B) ‘Likely functional ’—missing a single ADP/ATP contact; (C) ‘Less likely functional’—missing several ADP/ATP contacts, (D) ‘Uncertain’—missing a single motif; (E) ‘Likely not functional’—with several missing motifs and (F) ‘NBS fragment’ (Supplementary Table S2).

### NLRscape sequence clustering

The NLRscape sequences were grouped according to three main criteria: their domain layout, their homology and their taxonomical spread:

#### By domain organization layout

Sequences were grouped into 6 main types: CNL, TNL, RNL, NL, NBS-only and unclassified. Further on, the main CNL, TNL and RNL groups were further gathered according to their layout into: (a) canonical organization, (b) canonical core + marginal domains, (c) incomplete and (d) atypical. Moreover, a distinction was made in case of any variation on the expected subunits of the *proper NBS* (NBS_p_) domain layout (NBD-ARC1-ARC2), designating any incomplete layout, subunit ablation, shuffling or duplication as a potentially *improper NBS* (NBS_i_).

Whereas the canonical organization (a) CC/TIR/RPW8–NBS_p_–LRR is prevailing especially in CNL and RNL groups, frequently this layout occurs in the presence of additional marginal domains upstream or downstream of the canonical core. In this case, the NLR falls in the second category (b)—canonical core & marginal domains: *– CC/TIR/RPW8–NBS_p_–LRR–*. Here, the symbol * denotes any domain with sequence larger than 50 residues. As many of the sequences originate from transcriptomic data, entries displaying an incomplete NBS domain with the domain layout consistent with a canonical core (*–CC/TIR/RPW8–NBS_proper/incomplete_), were assigned as potentially incomplete sequences (c). Entries displaying any domain or subdomain shuffling, ablations or repetitions that are inconsistent with the canonical core organization were assigned to the *atypical* group (d). Around 15% of NLR sequences lack any of the canonical N-ter domains: CC, TIR or RPW8 but display an NBS-LRR layout. These were named NL and were found to fall into the following categories: (a) canonical NL core with or without marginal domains (*–NBS*p*–LRR–*), (b) incomplete NLs and (c) atypical NLs. Additionally, ∼12% of the NLRscape sequences lack both of the canonical N-ter and C-ter domains and are NBS-only with subdomains in either canonical or incomplete. Finally, around 2% of the sequences display NBS subdomains associated or not with non-canonical regions. There were assigned into a residual ‘Unclassified’ category.

Given that in the full set many sequences display unusual complex domain organization, with frequent atypical layouts which might result from potentially erroneous gene prediction we also retained separately a curated subset of 29 008 sequences—by selecting only those entries displaying layouts compliant with the canonical domain organization, containing all of the nine motifs essential for NBS stability and nucleotide binding and described in UniprotKB to having no current evidence for potential erroneous gene prediction.

#### By homology

Sequences of both full and curated sets were initially clustered at 90% sequences identity and 90% coverage thresholds, in order to group highly redundant sequences. The resulting cluster representatives were next funnelled into clusters with increasing identity from 30% to 70% in 10% increments at 70% overlap thresholds using MMseqs2 (Steinegger and Söding, 2017) based on a greedy set cover clustering method and a bidirectional overlap criterion.

Clusters at each identity threshold were next subjected to a bioinformatic analysis pipeline consisting in generating MSAs, identity/overlaps matrices, secondary structure, domain, motif consensuses and computing clusters graphs displaying inter-cluster homology relationships. MSAs were performed using Mafft (52), while the cluster sequence variability was computed using Logomaker (53) and expressed as Kullback–Leibler divergence. The secondary structure predictions were computed with RaptorX-property package (54,55) and LRR motifs with LRRpredictor (50) for each sequence of the clusters and used in raising cluster consensuses via a majority vote rule. Cluster graph analysis was performed using Cytoscape (56,57) defining clusters as nodes and identity percentages between cluster representatives as edge weight spring constant in a force-directed embedding.

#### By taxonomy spread

The resulting homology clusters at different identity/overlap thresholds were further classified according to their taxonomic spread on seven levels: order, supraorder, subclass, class, supraclass, phylum and kingdom. Clusters containing NLR sequences originating all from a single taxonomic order were classified as order-specific while the ones originating from at least two orders of the same class were classified as supraorder-specific, and so on hierarchically.

### NLRscape database and web interface implementation

The NLRscape database is organized as a MySQL relational database which can be downloaded either as a standalone database or as individual tsv/csv tables. The web interface is implemented using PHP, Javascript and Bootstrap to provide an easy-to-use responsive interface that facilitates data analysis and visualization and provides customized analysis tools. Interactive taxonomic sunburst diagrams, 1D and 2D histograms and heatmaps charts are implemented using the Plotly library (58), while the sequence visualization page uses ProtVista library (59) for mapping annotations on the protein sequence. Cluster graphs are displayed using the Cytoscape JS library (60). Synoptic ‘all-data’ plots are generated using in-house scripts which utilize LogoMaker (53) for entropy plots. For visualizing the 3D structures of available AlphaFold models (61,62) the 3Dmol.js (63) library is used, while for multiple sequence alignments synchronized with mapped predictions of secondary structure and motif annotations – two tools are implemented and customized: an in-house PHP viewer and the MSAviewer (64).

## RESULTS AND DISCUSSIONS

### Overview of NLRscape, an atlas of plant R proteins

#### NLRscape web interface structure

**Figure 1.**
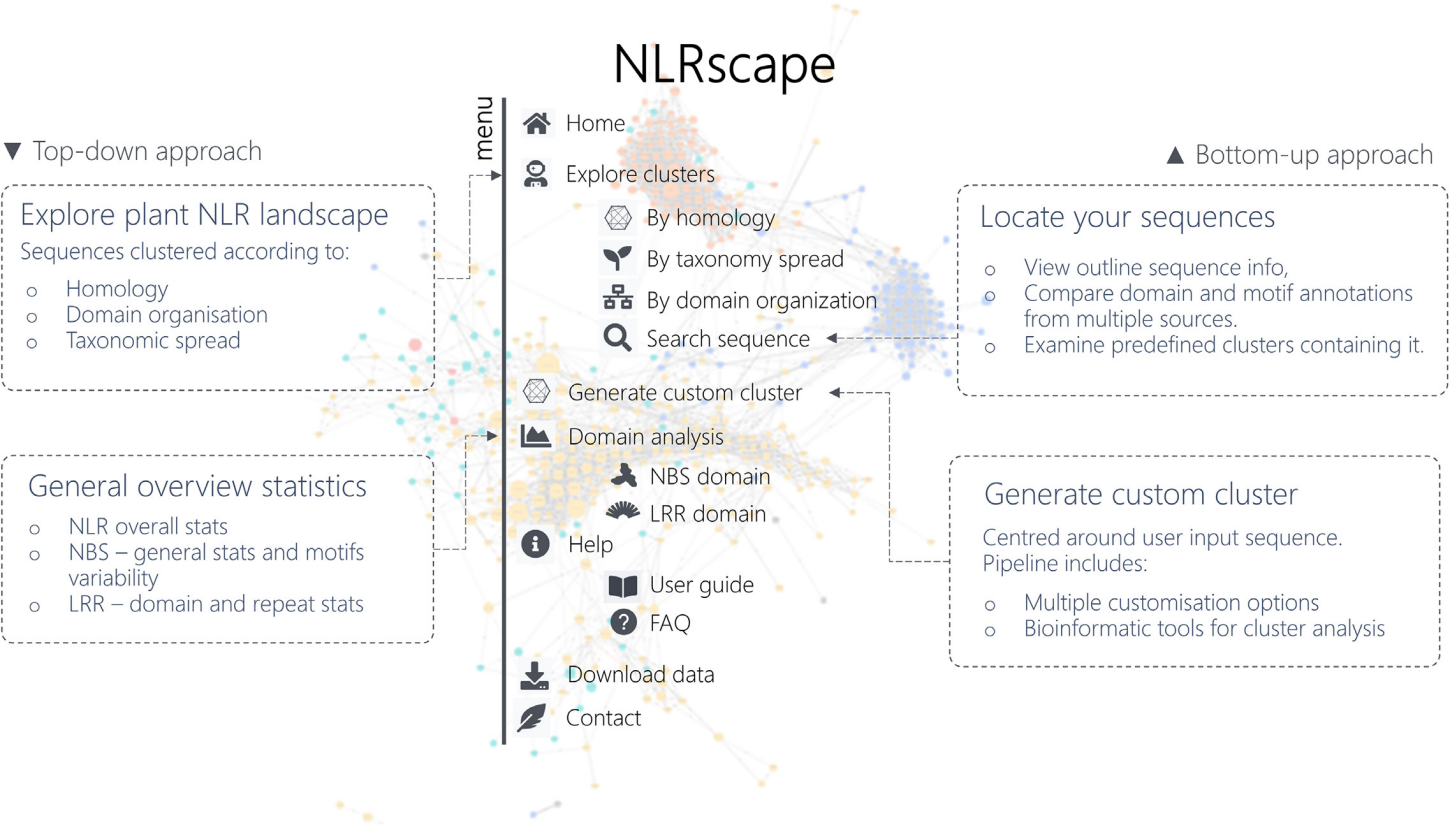
NLRscape atlas—a brief overview of the main features.

NLRscape is a webserver database that curates a collection of over 80 000 putative NLR sequences from the entire plant kingdom identified in UniProtKB to contain at least one NBS-like domain, as these activation switch domains are central to all NLR classes (65). For each entry, NLRscape provides annotations concerning both the overall domain organisation and the main known local motifs within canonical TIR/CC, NBS and LRR domains. NLRscape offers also a large number of bioinformatics and data visualisation tools aimed at the exploration of the complex sequence landscape of this class of receptors in plants. An overview of NLRscape main features and services is shown in Figure 1.

The ***Home page*** introduces users to the main features and latest updates of the NLRscape. It also provides a quick search engine allowing users to find sequences or groups of sequences according to various criteria such as sequence name, Uniprot ID, domain organization, organism etc. The ***Explore clusters*** menu allows the exploration of predefined clusters organizing the NLR sequences according to various criteria – homology, domain organization and taxonomic spread, facilitating a *top-down perspective* on the NLR landscape. Under ***Generate custom cluster*** NLRscape provides also services for a *bottom-up approach* allowing a user to either locate given known receptors in the grand NLR landscape and examine precomputed clusters; or, alternatively, allowing them to generate custom clusters centered around new NLR sequences and perform analyzes using the NLRscape tools. The ***Domain analysis*** menu provides for the time being overviews of NBS and LRR domain properties.

**Figure 2.**
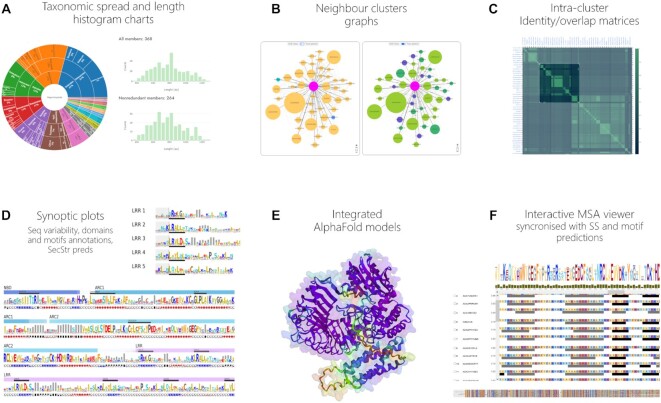
Tools for cluster analysis.

Amongst the interactive online tools provided by NLRscape for cluster analysis we mention: (a) taxonomy distribution plots and sequence length distribution; (b) cluster graphs for assessing sequence vicinities and homology relationships; (c) identity and overlap matrices between the cluster members; (d) synoptic plots of cluster sequence variability with mapped consensuses of secondary structure, domain and motif predictions; (e) 3D visualization of the available AlphaFold models; (f) interactive MSA synchronized secondary structure and motif predictions (Figure 2).

#### NLRscape entry sequence page

Each sequence entry page gathers summary information including UniProtKB ID and links to UniprotDB entry, protein and gene alternative names, organism origin and lineage, and links to the main publications describing its structure, properties and interactions. Protein evidence, sequence caution flags and the five-point annotation score from UniprotKB indicating potential incomplete or erroneous gene predictions are provided for quality control. External links are in addition provided to the mapped entries in other databases aiming to allow users to examine the original sequence genomic or transcriptomic source and to gain an overall picture on the level of cumulated knowledge of experimental exploration of each entry.

The introductory section is followed by an NBS domain integrity evaluation based on a 6-state score (A–F) describing the compliance of 9 NBS critical motifs and 15 ADP/ATP key contacts in order to provide a quick estimate over its potential functionality.

Next section provides a summarizing overview of the main information regarding—NLRscape, Interpo and UniProtKB domain and motif annotations, followed by additional relevant data from UniProtKB such as reported mutagenesis sites with links to the corresponding publication, sequence conflict information, proteomic data and variant annotations—mapped for each entry onto the amino acid sequence.

The sequence mapping section is followed by the Clusters section of the groups to which the entry was assigned. A link is also provided to generate custom clusters around the sequence at chosen identity and overlap cutoff levels and homologue taxonomic origin.

Whenever available for the given entry the AlphaFold model (62), is made available for visualization in the NLRscape bench and may be used to map cluster variability onto the 3D model—to analyze the emerging conserved patches and locate the more variable regions. This section is followed the sequence domain annotations comprising two tables—the first with the refined NLRscape annotation and the second with cross-reference annotations from various other sources. In addition, precomputed NLR-specific motif predictions using LRRpredictor (50) and NLRexpress (51) are provided.

### Organization of NLRscape sequence dataset

Users can examine the properties of a given NOD-like receptor family using provided bioinformatic tools and locate the sequence in the complex NLR landscape relying on precomputed NLR groups and clusters. To facilitate the exploration of the NLR families, sequences were grouped according to three main criteria: *domain organization*, *sequence homology* and *taxonomic spread*.

#### Grouping sequences according to their NLR domain layout

From a total of 80 300 NLRscpe sequences around 67 000 of them were found to comprise both NBS and at least another canonical NLR domain: CC, TIR, RPW8 or LRR. Based on the domain organization, NLRscape sequences were further classified into four main NLR groups: CNL, TNL, RNL, NL and two residual groups: ‘NBS only’ and ‘Unclassified’, summing up to a total of 2106 distinct domain layouts.

**Figure 3.**
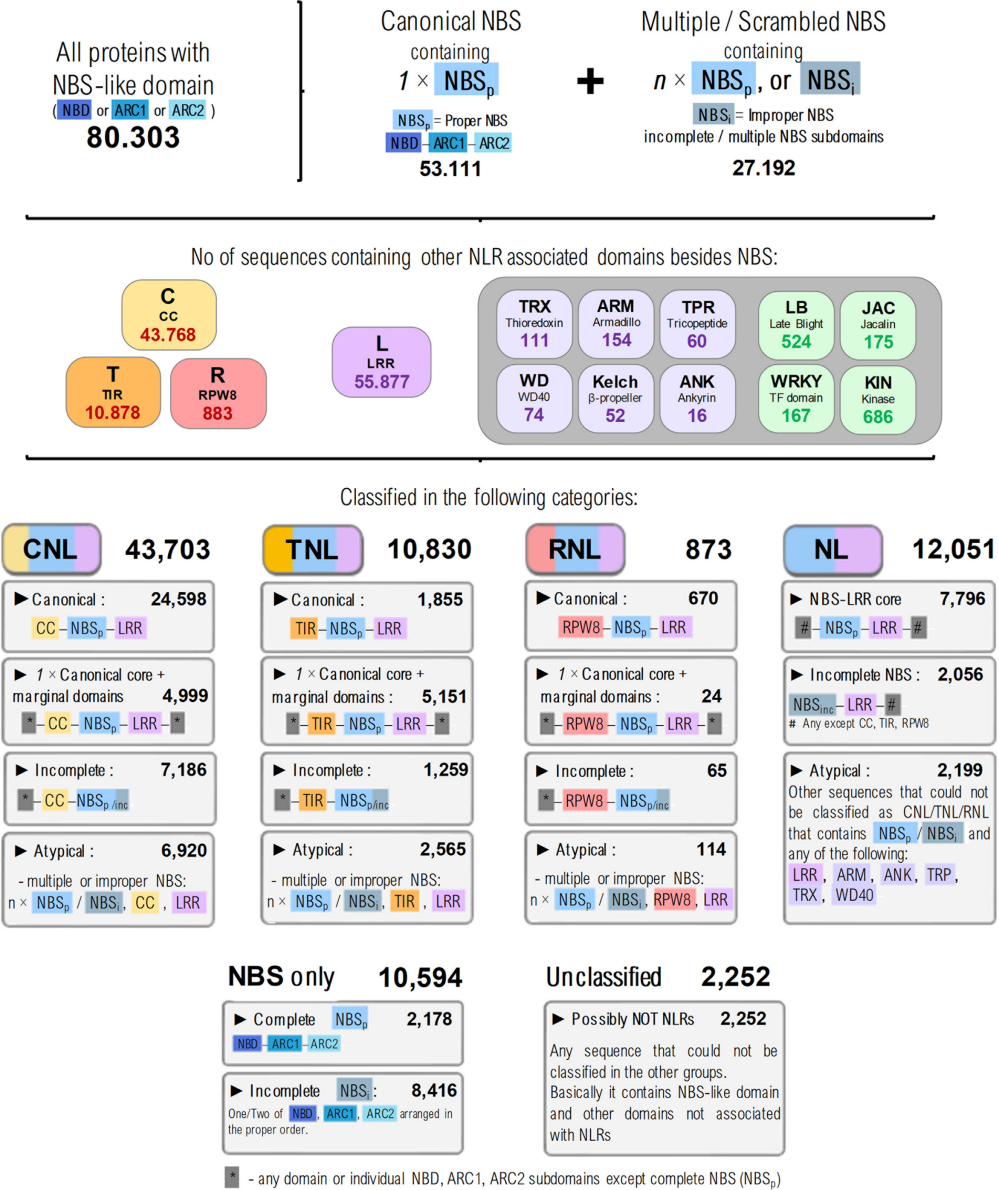
Overview of the main NLR classes and their domain organisation in NLRscape.

Interestingly, the domain configuration profiles of the collected sequences are more complex than initially expected with only around half of them showing regular CC/TIR/RPW8–NBS–LRR organization and the rest either displaying additional domains or showing domain/subdomain shuffling, ablation or duplication. Only ∼53k sequences contain a single proper NBS (NBS_p_) with the expected configuration NBD–ARC1–ARC2, while ∼27.2k sequences contain either multiple proper NBS domains or abscised/duplicated subdomains (Figure 3).

The CNL majority, 56%, shows a canonical organization while ∼11% contain a canonical core supplemented by additional marginal domains with the rest being either incomplete or atypical. By contrast, only 17% of the identified TNL sequences contain solely the canonical TIR–NBS–LRR organization, while 47% of them include additional domains mainly in the C-terminal region downstream LRR—one extra domain in 85% of cases. While much smaller than CNL and TNL, the RNL group shows the highest tendency for a simple, canonical RPW8-NBS-LRR organization, with only less than 25% displaying additional extra domains or incomplete/atypical layouts.

A small fraction (< 3%) of the marginal domains flanking the canonical core (CC/TIR/RPW8–NBS–LRR) consists of various mixtures of complete/incomplete NLR domains (additional CC/TIR/NBS/LRR domains) or entire canonical cores concatenated sequentially. Given their isolated occurrence, these cases could be explained by NLR genes’ close proximity in the genome, but their biological functional effect is yet uncharacterized.

While other solenoid-like domains besides LRR (such as ARM, ANK, TRP, WD40, Kelch, etc) have been reported so far (66) in NOD-like receptors, these sum up to <500 seq, in NLRscape dataset—compared to ∼55 900 seq with LRR domains, i.e. <0.1%. Also, many of the alternate solenoid domains are found together with the LRR in the same sequence.

The noncanonical NLR-like sequences were further classified into two groups: incomplete and atypical. The incomplete sequences show premature ends or N-ter ablations of a potential canonical core and this could be due to incomplete transcripts. Their occurrence is similar in CNL and TNL (11–17%) and lower in the RNL group (7%). The atypical group comprises all sequences that, despite containing the expected domains, their order is shuffled or contain duplicated domains/subdomains inside their core. While these could be explained by the vicinity of NLR genes, their biological effect is yet to be investigated. The fraction of atypical domain organization is variable between NLR groups—CNL: 16%, TNL: 23%, RNL: 13%.

Around 10.5k sequences appear to consist only in either incomplete fragments of NBS (∼8k) or encode a complete NBS (∼2k) without additional domains. A total of ∼2.5k proteins remained unclassified and contain NBS subdomains joined with other domains not associated with NLRs and were kept in the database for further sequence and structural analyses.

#### Grouping sequences by homology

**Figure 4.**
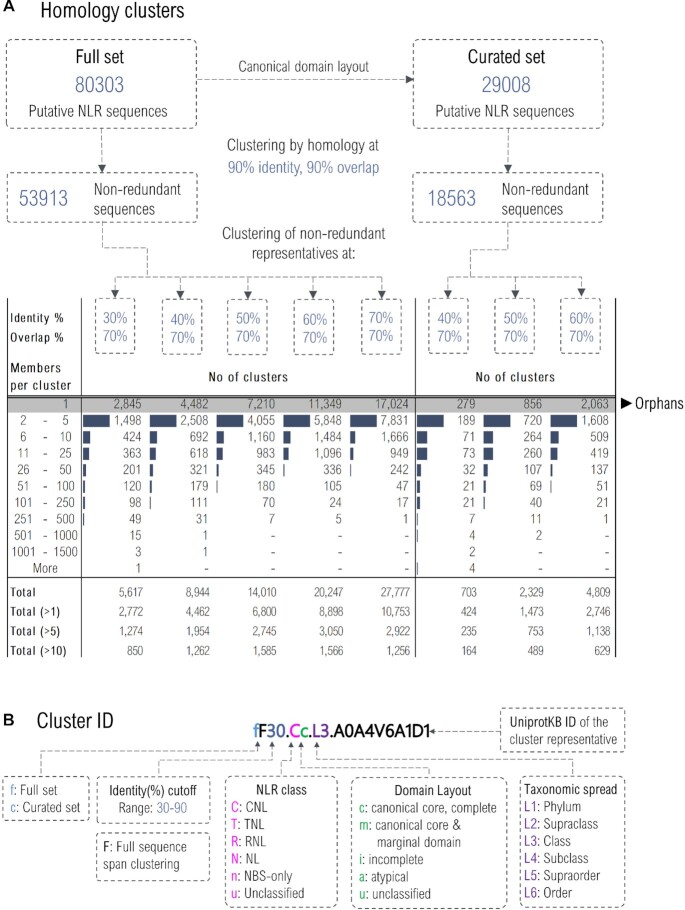
(**A**) Clustering by homology workflow alongside cluster member histograms for each identity cutoff. (**B**) Description of the cluster naming system.

The total of over 80 000 sequences, was initially subjected to an initial clustering at 90% sequence identity and 90% overlap in order to address highly redundant or identical sequences. The resulting ∼54 000 representatives at 90% identity were funnelled in clusters from 30% to 70% identity in increments of 10% using a 70% overlap cutoff and subjected to analysis as described in the methods section (Figure 4).

At all identity levels, orphans are dominant representing around half of the sequences while a quarter of the clusters are small with less than five members (Figure 4). This wide NLR spread in the sequence space might be due to many causes aside those resulting from the clustering procedure. Among these the transcriptomic origin of the data can not be excluded—as transcripts resulting from different exons with particular deletion structures could fall outside main clusters resulting in orphans. This is in line with recent genomic analysis showing that the NLR genes which are often grouped in large genome blocks are subjected to increased dynamics even between same species individuals (37,38). These orphan sequences could correspond to either (a) potential gene prediction artefacts, (b) incomplete transcripts with potentially impeded functionality, (c) ‘helper’ gene products or (d) NLRs containing bait domains which are not shared with close-by species—and to discriminate between these possible instances the NBS functionality score (A–F) might be of use for a preliminary assessment. However, as these outliers do not group with the canonical NLR they do not affect any cluster analysis. Moreover, as fringe molecular species these might be of real interest for hunting for more specific functions in innate immunity system. Orphans were therefore kept in the full set for groups interested in the detailed investigation of intra-species diversity, in contrast to the larger NLR clusters displaying members with broader taxonomic spread which are of interest in studying canonical resistance mechanisms shared by entire clades.

Given that most of the putative NLR sequences originate from gene predictions with only less than 1500 sequences supported by transcriptomic and/or proteomic data, a curated subset was next generated by selecting from the full set only those entries that: (a) are compliant with a canonical domain organization of the main NLR classes: CNL, TNL, RNL and NL; (b) possess, in the right order, all the nine NBS motifs essential for ADP/ATP switch function and (c) are not flagged in UniprotKB for potential gene prediction errors.

With around 29 000 sequences, this curated set comprises 36% of the full set and was also subjected to homology clustering at 40%, 50% and 60% identity thresholds (Figure 4). Results are provided in the online atlas as comparison of the full and curated clusters could be helpful in tracing gene prediction problems, especially in the case of unusual domain layouts. Nevertheless, even in this curated set caution should be taken with lower scored sequences as in the absence of transcriptomic and/or proteomic evidence, erroneous gene predictions such as missing/misplaced exons or donor/acceptor predicted sites cannot be excluded.

#### Grouping sequences by their taxonomic distribution

**Figure 5.**
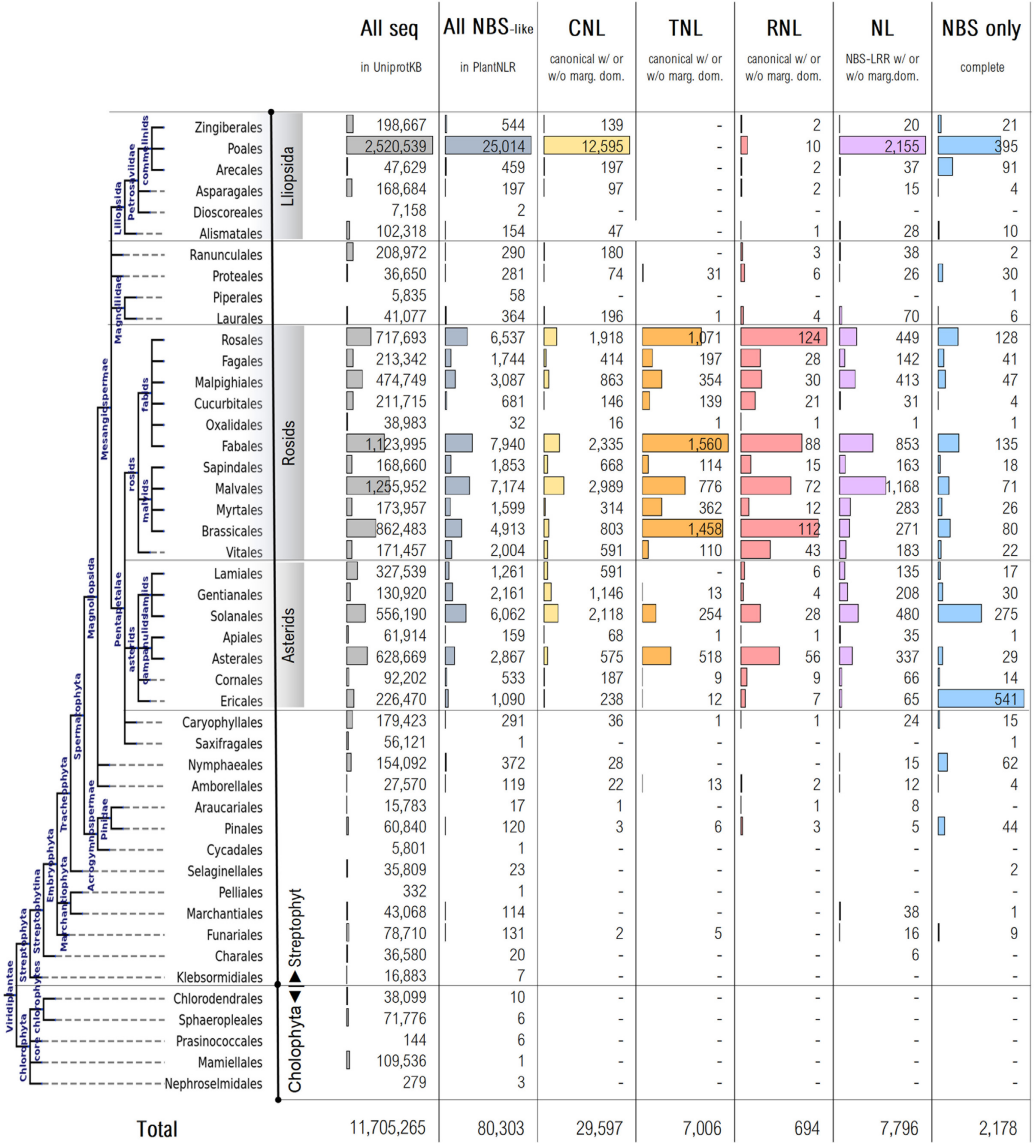
Taxonomic distribution of the main NLR classes. Included are sequences showing the complete canonical organisation with/without marginal domains. To ease comparison also provided is the baseline distribution computed on the entire UniProtKB database.

NLR layouts and clusters were also classified according to their taxonomic spread—for instance, a quarter of CNLs are found in monocots, in the *Poales* order. This mirrors the fact that this clade is also highly represented in general in UniProtKB with ∼2.5 million total sequences, while other monocot groups (in *Liliopsida*) are significantly less represented (such as *Dioscoreales* with a total of only ∼7k known sequences). Roughly, the CNL distribution in *Magnolipsida* class is similar to the background distribution of all known sequences in UniProtKB (Figure 5).

TNL sequences with complete canonical cores were not found in monocots, consistent with previous analyses (67). Most TNLs are present in rosids and asterids. Several TNLs were found in *Proteales* (lotuses), and outside flowering plants class: in *Amborellales*, *Pinales* and in *Funariales* (mosses).

The RNLs taxonomic spread is similar to TNLs, concentrated in asterids and rosids clades. Outside Pentapetalae, several RNLs were found in monocots (17 seq), *Ranculales* (3), *Proteales* (6) and *Laurales* (4) and outside flowering plants: in *Amborellales*, *Pinales* and *Araucariales* (a single seq), but not in mosses (*Funariales*).

The NL sequences with complete NBS-LRR core show a taxonomic distribution similar to the CNL group. Examples were found in more basal streptophytes which do not contain CNLs/TNLs/RNLs in *Marchantiales* (liverworts) and *Charales* (green algae). While several NBS containing proteins were found in the *Chlorophyta* phylum, these are atypical or incomplete and no sequences with complete canonical core were found.

In addition homology clusters at each identity threshold were classified according to their taxonomic spread on seven levels: order (L6), supraorder (L5), subclass (L4), class (L3), supraclass (L2), phylum (L1) and kingdom (L0). A 2D graph embedment was generated—depicting clusters as nodes and homology relationships as edges – in order to allow the analysis of how particular traits of an NLR class are scattered within the entire kingdom.

**Figure 6.**
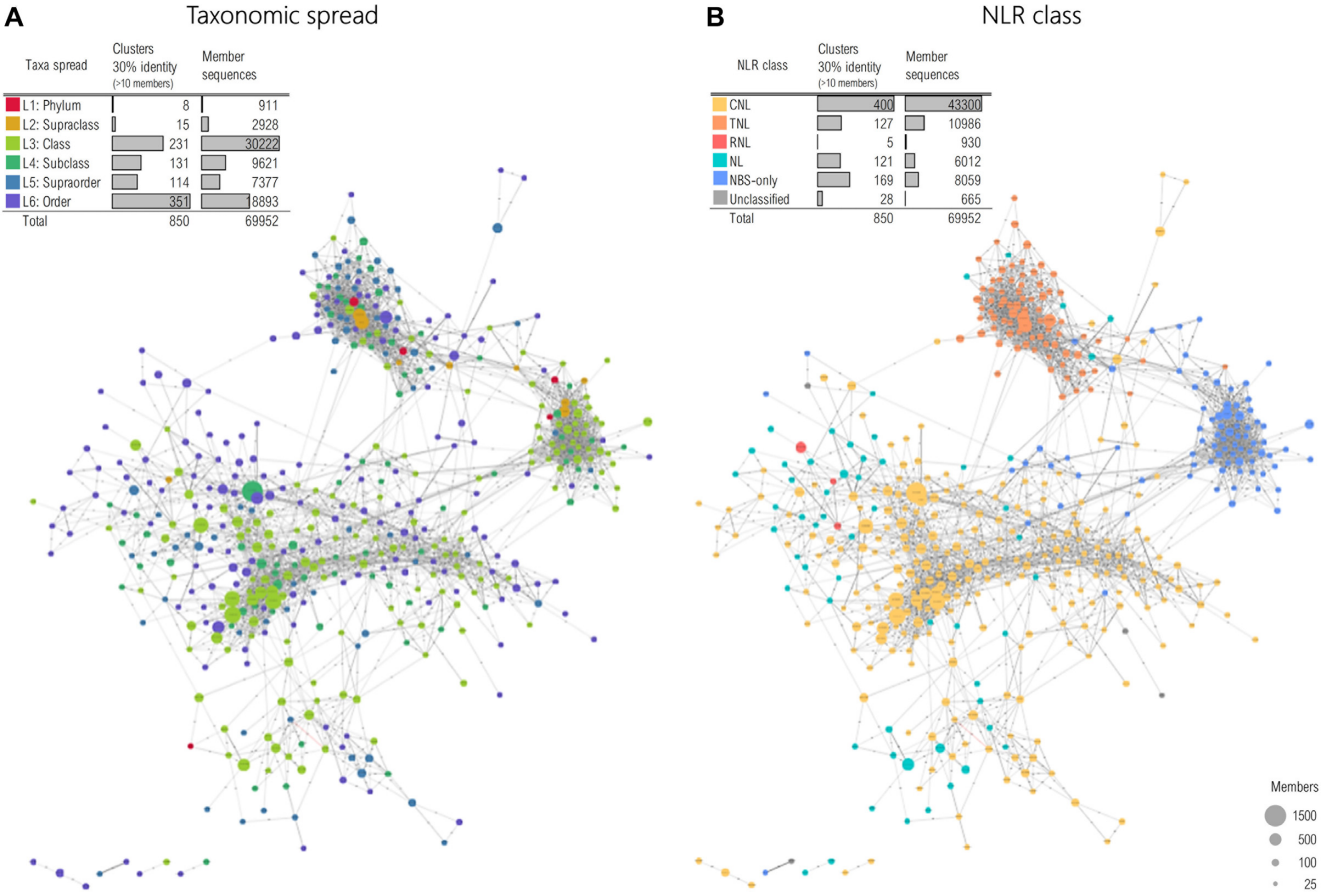
The taxonomic spread (**A**) and NLR class distribution (**B**) of the homology clusters at 30% identity. The 2D graph embedding represents clusters as nodes with a radius proportional to the number of constituents, coloured accordingly to the histogram, while edges illustrate inter-cluster homology relations. Displayed are only clusters with more than 10 members.

Zooming in on clusters at 30% identity (of >10 sequence members), computed on the full set, around 27% of the sequences are grouped in order-specific clusters, whereas half of them are in clusters with class spread (Figure 6A). While no cluster shows kingdom spread (L0), a small fraction (1%) of them have a broad (L1) phylum spread, but most of these consists of atypical or incomplete sequences.

As illustrated in Figure 6A, broad taxa clusters (L2/L3) are escorted by a slew of lower level ‘satellite’ clusters (L5/L6), which is consistent with current phylogenetic models describing the continuous and broad specialisation of NLR across species (7,68,69). On the other hand, Figure 6B indicates that according to the layout distribution, NLR classes are tightly coupled, with homology relations between clusters—with CNL, TNL and NBS-only groups clearly separated by the homology graph embeddings (Figure 6B). Interestingly the tighter wrapping of TNLs compared to the more scattered CNL region of the graph is consistent with previous studies (70) indicating an increased evolutionary dynamics of CNL genes compared to TNLs.

## CONCLUSIONS AND PERSPECTIVES

The herein presented atlas of plant NOD-like receptors gathers information on over 80 000 putative NLR protein sequences organized in clusters according to their domain layout, homology and taxonomic spread. The webserver provides a series of interactive online bioinformatic tools aimed at facilitating an overview of the natural diversity of the plant NLR family one of the largest, highly diverse and most studied group of plant proteins—given their economic importance in crop breeding for disease resistance.

On this line, NLRscape aims to complement with a more structural approach at the protein sequence level, other public resources dedicated to plant immunity such as PRGdb, RefPlantNLR (4,5) which are mainly focused on disease resistance and genomic data analyses and annotations, NLR gene identification or gene orthology. Besides the synoptic, ‘atlas’ view of the NLR landscape, the webserver facilitates also a bottom-up approach. Starting from a given input consisting in a new or already known NLR sequence—users can generate custom clusters and perform structural analyses centered around the provided sequence—which might be potentially useful in bioengineering and artificial evolution attempts. Such type of sequence to structure mapping analysis was used for instance to convert virus into nematode resistance (35) or to predict the location in sequence of the NBS-LRR interface (43) long before its cryo-EM identification in ZAR1 (28,29), or to formulate hypothesis on pathogen-sensing receptors based on LRR surface amino acid conservation (37). Examples on how clustering combined with structural annotation can be used to help experimental research are also provided by NLRscape under the Help section.

Up-coming releases will include additional bioinformatic analysis tools such as sequence correlation analysis tools, phylogeny analysis tools and variability mapping onto the 3D structures. Further releases will also address: (a) problems related to automatic update of NLRscape; (b) a thorough analysis and clusterisation system of the canonical NLR domains: CC/TIR/RPW8, NBS & LRR and (c) wherever possible add annotations related to the non-canonical regions of NOD-like receptors which are notated here generically [*] but may contain enlightening information regarding the function and evolutionary dynamics of this protein family.

While in its current form NLRscape covers information on only plant NOD-like receptors, further developments aim to expand the database and analysis tools to fungi and animal NLRs. Online instruments meant to examine similarities and differences between plant/fungi/animal innate immunity will be of interest to a wider audience involved in the investigation of sequence-structure-function relations in this critically important class of innate immunity proteins. In this way combining recent advances in big-data, structural and computational biology will clearly help our understanding of how interaction with pathogens had shaped the NLRs sequence landscape and generated the large repertoire of immune-related functions and also provide us with a framework for bioengineering, not only with applications in agriculture but also in medicine.

## DATA AVAILABILITY

The NLRscape webserver and raw database can be found at: https://nlrscape.biochim.ro/.

## Supplementary Material

gkac1014_Supplemental_File
