# Supplementary material for: NLRscape: an atlas of plant NLR proteins
Source: Nucleic Acids Res. 2022 Nov 9;51(D1):D1470–82. doi: 10.1093/nar/gkac1014 (PMC9825502; doi:10.1093/nar/gkac1014)
Supplement: gkac1014_Supplemental_File [file gkac1014_supplemental_file.pdf]

Supplementary material

Supplementary Table 1

NBS motifs	NBS subdomain	Motif role	Motif consensus	Considered ADP/ATP contacts
**VG-motif**	NBD	ADP/ATP-pocket	bbGRE	-
**P-loop**	NBD	ADP/ATP-pocket	GbGGbGKTT	G0, G3, G5, K6, T7
**RNBS-A**	NBD	Structural	FDbrhWbsbs	
**Walker-B**	NBD	ADP/ATP-pocket & structural	KRbbbbDD	D7
**RNBS-B**	NBD	ADP/ATP-pocket & structural	KbbbTTR	T4, R6
**RNBS-C**	ARC1 / HD	ADP/ATP-pocket	bseeeSWebF	b0, b8
**GLPL**	ARC1 / HD	ADP/ATP-pocket	GLPLA	P2, L3
**RNBS-D**	ARC2 / WHD	Structural / contact interface	CFLYCSLFP	-
**MHD**	ARC2 / WHD	ADP/ATP-pocket	bHD	b0, H1, D2

Supplementary Table 1. The NBS sequence motifs and ADP/ATP contacts considered in scoring NBS domains

Supplementary Table 2

NBS status	NBS motifs compliance (9 motifs)	ADP/ATP contacts compliance (15 total contacts)
● **Highly likely functional**	9 out of 9	15 out of 15
● **Likely functional**	9 out of 9	14 out of 15
● **Less likely functional**	9 out of 9	⩽13 out of 15
● **Uncertain**	8 out of 9	
● **Likely not functional**	3-7 out of 9	
● **NBS fragment**	< 3 out of 9	

Supplementary Table 2. Classes of the NBS integrity scoring and utilised criteria.
